# Building a better model of the retina

**DOI:** 10.7554/eLife.51183

**Published:** 2019-10-04

**Authors:** Milica Radisic

**Affiliations:** 1 Department of Chemical Engineering and Applied ChemistryUniversity of TorontoTorontoCanada; 2 Institute of Biomaterials and Biomedical EngineeringUniversity of TorontoTorontoCanada; 3 Toronto General Research InstituteUniversity of TorontoTorontoCanada

**Keywords:** retinal organoids, retinal pigment epithelial cell, human induced pluripotent stem cells, bioengineering, None

## Abstract

Researchers have combined organ-on-a-chip engineering with the benefits of organoids to make improved models of the human retina.

**Related research article** Achberger K, Probst C, Haderspeck J, Bolz S, Rogal J, Chuchuy J, Nikolova M, Cora V, Antkowiak L, Haq W, Shen N, Schenke-Layland K, Ueffing M, Liebau S, Loskill P. 2019. Merging organoid and organ-on-a-chip technology to generate complex multi-layer tissue models in a human retina-on-a-chip platform. *eLife* **8**:e46188. doi: 10.7554/eLife.46188

A lot of what is known about human cells has come from studying cultures that have been grown in a dish. However, these two-dimensional cultures do not accurately represent the native environment in which our cells grow. Over the past decade both bioengineers and stem cell biologists have created three-dimensional cell cultures that promise to provide a more reliable model for studying human tissue. Bioengineers, for example, have been creating ‘organ-on-a-chip’ devices that precisely control the various cues that act on cells (Huh et al., 2010; Zhang et al., 2016). Using these devices, bioengineers have been able to reproduce the three-dimensional environments of certain tissues, including heart and lung tissues, in a way that captures some aspects of how these organs work (Zhang et al., 2018).

Stem cell biologists, on the other hand, have relied on the inherent ability of stem cells to undergo differentiation in a three-dimensional environment, and to create cell masses called organoids that exhibit some complexity of native organs, including the brain (Lancaster et al., 2013; Lancaster and Knoblich, 2014) and kidney (Phipson et al., 2019). There is no doubt that organoids provide a more faithful representation of native tissue than organ-on-a-chip devices, which typically contain at most two to three different cell types. However, organoids exhibit a lot of variability and this, along with their small size, means they cannot easily be used to study how an organ may respond to different drugs. Although organ-on-a-chip devices are generally considered to be less biologically representative, they do offer a more controlled environment for studying the effects of drugs. Therefore, the natural way forward for both these fields would be to combine the advantages of organoids and organ-on-a-chip devices in a single system (Takebe et al., 2017).

Human vision is particularly difficult to study using animal models, since the fundamental characteristics of how we see are different to most animals. This has motivated researchers to use models of the human retina that have been explanted post-mortem, but these can display a lot of donor-donor variability and various disease-related conditions. Recently, researchers have used pluripotent stem cells to generate an organoid of the human retina that has a similar structure to the real thing (Zhong et al., 2014). However, for these models to accurately represent the in vivo environment of the retina, they must also have a blood supply that provides the retina with nutrients. Now, in eLife, Peter Loskill (Fraunhofer Institute and University of Tübingen), Stefan Liebau (University of Tübingen) and colleagues – including Kevin Achberger, Christopher Probst and Jasmin Haderspeck – report the creation of a retina-on-a chip device that reproduces the vascular supply to the retina in vitro using specially designed micro-channels (Achberger et al., 2019).

To create the device, Achberger et al. combined organoids of the human retina with retinal pigment epithelial (RPE) cells, which form part of the complex environment of the human retina in vivo. Each chip has four compartments designed to hold a single organoid, which sit above a porous membrane that separates the retina from the vasculature-like compartment below (Figure 1): First, RPE cells were seeded onto the membrane, followed by the organoids which had been cultured for 180 days prior to plating to ensure they had reached maturity. This design allows small molecules, nutrients, and proteins from the media in the vascular-like channels to diffuse across the porous membrane where they can be transported from the retinal epithelium into the organoids, similar to how transport occurs in vivo. Secreted factors are then carried away from the retina tissue via the flow of fluids which are confined to the vasculature-like channels below.

**Figure 1.**
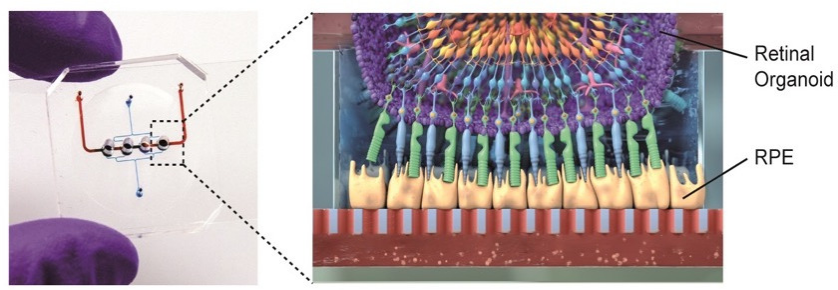
Retina-on-a-chip combines the benefits of organoids and organ-on-a-chip engineering. Each chip has a vascular-like compartment (red) which is separated from the four organoid compartments above by a porous membrane. Schematic diagram (right) showing the various layers of cells in the retinal organoid (top) and the retinal pigment epithelium cells (RPE; yellow).

Image credit: Achberger et al. (CC BY 4.0)

This retina-on-a-chip device generated by Achberger et al. is the first tissue model to reproduce the specific layer configuration of seven different cell types in the human retina. The organoids seeded into the device expressed markers of major retinal cells such as ganglion cells, bipolar cells, horizontal cells, amacrine cells, Müller glia and photoreceptors. Notably, Achberger et al. showed that RPE cells directly interacted with the outer segment of photoreceptors in the retinal organoid through phagocytosis. They also observed that cells in the organoid formed specific layers that were similar to how retinal cells are structurally organized in vivo (Figure 1). Achberger et al. also found that these devices could reproduce the visual side-effects of the anti-malaria drug choroquine and the antibiotic gentamicin, indicating that these retina models could be used for drug testing.

The retina is an especially difficult organ to model due to the large number of different cell types involved and its complex structure, so the device created by Achberger et al. is at the very forefront of the organ-on-a-chip field. Further studies should focus on: enhancing the throughput of device production and cell cultivation, automating the plating of cells and measurements taken, and eliminating the use of drug absorbing materials. The new device promises to decrease the reliance on animal models in retinal drug testing and could be used to study the effects of new drugs on human retinas before starting clinical trials. This retina model could therefore have a huge impact on drug discovery and safety testing.
